# Redução na Ativação Plaquetária: Um Potencial Efeito Benéfico do Exercício Regular na Doença Arterial Coronariana

**DOI:** 10.36660/abc.20201198

**Published:** 2021-03-03

**Affiliations:** 1 Clínica de Medicina do Exercício Rio de JaneiroRJ Brasil Clínica de Medicina do Exercício – CLINIMEX, Rio de Janeiro, RJ – Brasil.

**Keywords:** Doença de Artéria Coronariana, Doenças Cardiovasculares, Exercício, Atividade Física, Reabilitação, Plaquetas

A reabilitação cardíaca baseada em exercício (RCBE) leva a reduções significativas na mortalidade e no risco de eventos cardiovasculares adversos em pacientes com doença arterial coronariana (DAC).^1^^,^^2^ Como resultado, a RCBE é atualmente uma intervenção Classe 1A para prevenção secundária em DAC.^3^ Entretanto, apesar de esforços consideráveis em pesquisa nas últimas décadas, os mecanismos multidimensionais associados aos efeitos cardioprotetores do exercício físico regular ainda não estão totalmente elucidados.^4^

Por outro lado, o aumento da atividade plaquetária tem sido cada vez mais reconhecido como um elemento-chave na patogênese e progressão da aterosclerose. Através da liberação de citocinas e quimiocinas, as plaquetas ativadas realizam a função de mediadoras do recrutamento de leucócitos para o endotélio vascular, favorecendo não apenas a ruptura da placa e trombose, mas também contribuindo para as etapas iniciais da aterosclerose e para os estágios subsequentes da progressão da placa aterosclerótica.^5^

Portanto, um interesse especial foi despertado em relação ao papel do exercício físico na função plaquetária, e resultados conflitantes foram observados. Embora sessões agudas de exercícios extenuantes pareçam aumentar a ativação plaquetária e, portanto, aumentar o risco de eventos trombóticos, a prática regular de exercício físico demonstrou diminuir a adesão e agregação plaquetárias, possivelmente contribuindo para a redução do risco aterotrombótico observada em indivíduos fisicamente ativos.^6^^,^^7^

Para avançar no conhecimento sobre esse assunto, na edição atual dos Arquivos Brasileiros de Cardiologia, Durmuş et al.^8^ relatam os efeitos de um programa de RCBE na ativação plaquetária em pacientes com DAC estável. O volume plaquetário médio (VPM) foi avaliado no início do estudo e após seis semanas, e os valores basal e final foram comparados de acordo com a participação no programa de RCBE. Os autores observaram que, enquanto os não participantes exibiram uma redução não significativa do VPM (8,7 vs. 8,6 fL), uma redução de 13% no VPM foi vista nos participantes que completaram o programa de RCBE (9,1 vs. 7,9 fL) (p <0,01). Além disso, foi observada uma forte correlação positiva entre a variação do VPM e a participação na RCBE (r = 0,75). Portanto, os autores concluíram que essa diminuição no VPM observada com a participação na RCBE pode desempenhar um papel importante na redução do risco trombótico em pacientes com DAC estável.

Esses resultados significativos encontrados por Durmuş et al.^8^ entretanto, requerem alguma cautela. O tamanho das plaquetas, quando medido pelo VPM, demonstrou ser um marcador da função plaquetária e foi positivamente associado aos indicadores de atividade plaquetária.^9^ Portanto, o VPM é considerado uma ferramenta valiosa para a avaliação da ativação das plaquetas. No entanto, a análise e interpretação do VPM não é simples. Uma série de variáveis pré-analíticas e analíticas, incluindo o tempo entre a coleta de sangue e a análise e a temperatura de armazenamento da amostra, são conhecidas por afetar significativamente as medidas do VPM e, como tal, representam desvantagens importantes ao usar o VPM como uma medida indireta da atividade plaquetária.^10^ Portanto, mais estudos utilizando marcadores padronizados e mais confiáveis de ativação plaquetária devem ser realizados para superar essas questões metodológicas e confirmar os resultados relatados.

Em conclusão, o estudo conduzido por Durmuş et al.^8^ abre um novo caminho para pesquisas futuras com o objetivo de expandir nossa compreensão sobre o efeito da prática de exercício físico regular no comportamento funcional das plaquetas e o potencial mecanismo protetor induzido pelo exercício em pacientes com DAC que participam de programas de RCBE.
