# Supplementary material for: Redução na Ativação Plaquetária: Um Potencial Efeito Benéfico do Exercício Regular na Doença Arterial Coronariana
Source: Arq Bras Cardiol. 2021 Mar 3;116(3):441–2. [Article in Portuguese] doi: 10.36660/abc.20201198 (PMC8159569; doi:10.36660/abc.20201198)
Supplement: Translation

# Translation: Reduction in Platelet Activation: A Potential Mechanistic Link between Regular Exercise and Its Benefits for Coronary Artery Disease

**Affiliations:** 1 Rio de JaneiroRJ Brazil Clínica de Medicina do Exercício – CLINIMEX, Rio de Janeiro, RJ – Brazil.

**Keywords:** Coronary Artery Disease, Cardiovascular Diseases, Exercise, Physical Activity, Rehabilitation, Blood Platelets

Exercise-based cardiac rehabilitation (CR) leads to significant reductions in mortality and risk for adverse cardiovascular events in coronary artery disease (CAD) patients.^1^^,^^2^ As a result, exercise-based CR is currently a Class 1A intervention for secondary prevention in CAD.^3^ However, despite considerable research efforts in the past several decades, the multidimensional mechanisms associated with the cardioprotective effects of regular exercise are still not fully elucidated.^4^

On the other hand, increased platelet activity has been increasingly recognized as a key player in the pathogenesis and progression of atherosclerosis. Through the release of cytokines and chemokines, activated platelets mediate the recruitment of leukocytes to the vascular endothelium, favoring not only plaque rupture and thrombosis, but also contributing to the early steps of atherosclerosis and the later stages of atheroprogression.^5^

Therefore, special interest has been raised regarding the role of exercise in platelet function, and conflicting results have been observed. While acute bouts of strenuous exercise seem to enhance platelet activation, and, therefore, increase the risk of thrombotic events, engaging in regular exercise has been shown to decrease platelet adhesion and aggregation, possibly contributing to the reduced atherothrombotic risk observed in physically active individuals.^6^^,^^7^

To shed light on this topic, in the current issue of *Arquivos Brasileiros de Cardiologia*, Durmuş et al.^8^ report the effects of an exercise-based CR program on platelet activation in stable CAD patients. Mean platelet volume (MPV) was assessed at baseline and after six weeks, and pre- and post-assessment values were compared according to participation in the CR program. The authors found that, while non-participants exhibited a non-significant MPV reduction (8.7 vs. 8.6 fL), a 13% reduction in MPV was observed in participants that completed the CR program (9.1 vs. 7.9 fL) (p<0.01). Moreover, a strong positive correlation was observed between MPV variation and CR participation (r=0.75). Therefore, the authors concluded that this decrease in MPV seen with CR participation might play an important role in reducing thrombotic risk in patients with stable CAD.

These significant results observed by Durmuş et al.^8^ however, require a note of caution. Platelet size, when measured as MPV, has been shown to be a marker of platelet function and has been positively associated with platelet activity indicators.^9^ Therefore, MPV is considered a valuable tool for the assessment of platelet activation. Yet, the analysis and interpretation of MPV is not straightforward. A number of pre-analytical and analytical variables, including the time between blood collection and analysis and specimen storage temperature, are known to significantly affect MPV measurements, and, as such, they represent important drawbacks when using MPV as an indirect measurement of platelet activity.^10^ Therefore, further studies using standardized and more reliable platelet activation markers should be undertaken to overcome these methodological issues and confirm the reported results.

In conclusion, the study conducted by Durmuş et al.^8^ opens a new avenue for future research aiming to expand our understanding on the effect of regular exercise on platelet functional behavior and the potential mechanism of exercise-induced protection in CAD patients who engage in CR programs.
